# Dementia Friendly communities (DFCs) to improve quality of life for people with dementia: a realist review

**DOI:** 10.1186/s12877-024-05343-0

**Published:** 2024-09-20

**Authors:** Stephanie Craig, Peter O’ Halloran, Gary Mitchell, Patrick Stark, Christine Brown Wilson

**Affiliations:** School of Nursing and Midwifery, Queen’s University Belfast, Belfast, Northern Ireland

**Keywords:** Realist review, Realist synthesis, Dementia-friendly communities, Dementia friends, Dementia, Quality of Life

## Abstract

**Background:**

Currently, there are more than 55 million people living with dementia worldwide. Supporting people with dementia to live as independently as possible in their communities is a global public health objective. There is limited research exploring the implementation of such interventions in the community context. The aim of the review was to create and refine programme theory – in the form of context mechanism-outcome configurations – on how the characteristics of dementia-friendly communities (DFCs) as geographical locations interact with their social and organisational contexts to understand what works for whom and why.

**Methods:**

This realist review sourced literature from 5 electronic databases: Cochrane Library, CINAHL, Medline, Scopus, PsychINFO and Google Scholar, as well as relevant websites such as Alzheimer’s Society to identify grey literature. Methodological rigour was assessed using the Joanna Briggs Institute critical appraisal tool.

**Results:**

Seven papers were included in this realist review that focused on DFCs in a geographical context The implementation of DFC interventions emerged as a process characterised by two pivotal implementation phases, intricately linked with sub-interventions. The first intervention, termed Hierarchy Commitment (I1a/b), involves the formalisation of agreements by businesses and organizations, along with the implementation of dementia-friendly action plans. Additionally, Educational Resources (I1c) play a significant role in this phase, engaging individuals with dementia and their caregivers in educational initiatives. The second phase, Geographical/Environmental Requirements (I2), encompasses the establishment of effective dementia-friendly signage, accessible meeting places, and community support.

**Conclusions:**

This realist review highlighted a theoretical framework that might guide the development of dementia-friendly communities to enhance the experiences of individuals with dementia and their caregivers within DFCs. Emphasising the need for a theoretical framework in developing geographical DFCs, the review outlines contextual elements, mechanisms, and outcomes, providing a foundation for future studies. The ultimate goal is to establish a robust body of evidence for the sustainable implementation of dementia-friendly communities, thereby improving the quality of life for those with dementia.

**Study registration:**

This study is registered as PROSPERO 2022 CRD42022317784.

## Introduction

Currently, there are more than 55 million people living with dementia worldwide [1]. It is estimated that this number will rise to 139 million by 2050. Dementia is the seventh leading cause of death and one of the major causes of disability and dependence among older people globally, resulting in reduced quality of life for people with dementia and their care partners, with associated social and financial consequences [1].

Neurological changes that occur with dementia cause the individual to experience impairments; however, it is increasingly recognised that it is the intersection of these impairments with the physical and social environments encountered that creates the experience of disability for the person with dementia [2]. Since most people who have dementia live in communities, the structure and culture of those communities are likely to have an impact on how dementia is perceived [3]. In response to this, the World Health Organisation, Dementia Alliance International, and Alzheimer’s Disease International have created programmes that promote a community model of social participation [4].

People with dementia, as well as their families and carers, value meaningful connections [5, 6] and need to be active participants in their social networks to maintain meaningful social connections [7]. Supporting people with dementia and their carers to live as independently as possible in their communities by providing social and emotional support is a global public health objective [8]. The worldwide action plan on the public health response to dementia was adopted by the World Health Organisation (WHO) in May 2017 [8, 9]. The plan suggests that increasing public awareness and understanding of dementia and making the environment dementia-friendly will enable people with dementia to maximise their autonomy through improved social participation [10].

ADI [3] define a dementia-friendly community (DFC) as a place or culture in which people with dementia and their care partners can feel empowered, supported, and included in society- Table 1 identifies the main elements of a DFC.

**Table 1:** Main elements of a DFC defined by ADI [3]

1.	**People:**	People living with dementia must be included and able to actively contribute to their communities.
2.	**Communities**:	Physical and social environments must be appropriate to meet the needs of people with dementia.
3.	**Organisations**:	Businesses, organisations and services within a community must develop dementia-friendly approaches to facilitate engagement by people with dementia.
4.	**Partnerships**	Cross-sectoral support and collective action amongst all parts of a community to drive positive change.

While a community is typically characterised by its geographic location, communities can also be made up of people who have similar hobbies, religious affiliations, or ethnic backgrounds e.g., organisations with a specific focus of dementia- friendliness [3]. According to Lin and Lewis [11], the idea of dementia-friendly communities focuses on the lived experiences of individuals with dementia and is most pertinent to addressing both their needs and the needs of those who live with and support them. According to Mitchell, Burton, and Raman [12], dementia-friendly communities are likely to be all-inclusive and promote community engagement for everyone, not only those who have dementia.

Several models and frameworks have been developed to operationalise DFCs. The Dementia Friends USA Framework [13] focuses on raising awareness and understanding of dementia across various sectors. The Alzheimer’s Society in the UK [14] has a model emphasising awareness, participation, and stakeholder involvement. The Community Engagement Model prioritises the involvement of people with dementia and their caregivers in developing DFC initiatives. Social Inclusion Strategies aim to improve social inclusion through supportive environments and community education [15]. The Multi-Sector Collaboration Model promotes cooperation among local governments, healthcare providers, businesses, and other organisations to support people with dementia comprehensively.

The DFC concept is inspired by the World Health Organisation’s Age-Friendly Cities initiative [15, 16], which aims to create inclusive environments supporting active and healthy aging [17, 18]. Both dementia-friendly and age-friendly approaches emphasise empowering local stakeholders to enhance social inclusion, reduce stigma, and remove barriers in physical and social environments [19].

Despite its potential, the DFC concept faces challenges and criticisms. Swaffer [20] highlights that the language around dementia often perpetuates stigma, negatively impacting those affected. Swaffer [20] and Rahman & Swaffer [21] criticise many DFC initiatives as tokenistic, often failing to genuinely include people with dementia in decision-making. They advocate for an assets-based approach, recognising and leveraging the strengths of individuals with dementia. Shakespeare et al. [22] emphasise the need for a human rights framework to ensure dignity, respect, and full inclusion for people with dementia. Effective DFCs should go beyond superficial friendliness to ensure authentic inclusion, empowerment, and adherence to a rights-based approach.

### Background

Person-centered care is a foundational approach that emphasises treating individuals with dementia with respect, valuing their uniqueness, and understanding their behaviours as meaningful communication [23]. The bio-psychosocial approach provides a holistic framework [24], recognising dementia as influenced by biological, psychological, and social factors, guiding comprehensive care strategies. Attachment theory [25] offers insights into the behaviours and relationships of individuals with dementia based on their attachment histories. The need-driven dementia-compromised behaviour model [26] shifts focus to addressing underlying needs behind behavioural symptoms rather than merely managing them. Thijssen and colleagues’ work on social health and dementia-friendly communities [27] aligns well with these person-centered and psychosocial approaches, emphasising social participation, autonomy, and environmental adaptation. Key principles for dementia-friendly communities derived from these theories include recognising individuality, fostering supportive environments, promoting autonomy and meaningful engagement, interpreting behaviours as expressions of needs, and prioritising holistic health and positive relationships. Implementing these principles can enhance inclusivity and support for people with dementia, with ongoing evaluation and adaptation crucial for sustained effectiveness of dementia-friendly initiatives [28, 29].

The existing body of evidence offers support for the effectiveness of DFCs, with previous research exploring various dimensions of their establishment. One perspective underscores the significance of a robust policy framework and an enhanced support infrastructure [30, 31]. Alternatively, other studies delve into the priorities of individuals with dementia and their caregivers, emphasising factors such as fostering social connections and promoting acceptance of dementia within the community [4, 15, 32, 33]. Additionally, investigations into the experiences of people with dementia residing in DFCs, including their awareness of living in such a community, have been conducted [34].

Despite extensive efforts to evaluate DFCs, their effectiveness remains challenging to ascertain due to the multifaceted and complex nature of the intervention. The evaluation process is further complicated by the diverse needs and preferences of individuals with dementia, variations in resources and support across different communities, and the dynamic nature of dementia care and research. A recent rapid-realist review by Thijssen et al. [27] comprehensively examined how dementia-friendly initiatives (DFIs) function for people with dementia and their caregivers. While some studies have reviewed dementia-friendly hospital settings, such as Lin [35] and a realist review by Handley [36] Thijssen et al.‘s [27] rapid realist review primarily focused on initiatives often serving as building blocks in DFC development. These initiatives are typically activity-based and on a smaller scale compared to larger communities. Despite these valuable insights, there remains a limited understanding of how geographical DFCs specifically contribute to improving the quality of life for individuals living with dementia.

Dementia-friendly communities are complex interventions. Understanding what works, why and what factors help or hinder their effectiveness can optimise the design and implementation of DFCs for the benefit of individuals with dementia and their caregivers [37], thus contributing to the development of robust and impactful DFC interventions [38].

DFCs are often understood primarily as geographical communities, which has several important implications [30]. Defining DFCs geographically allows for a localised approach tailored to specific towns, cities, or regions, enabling initiatives to address the unique needs and characteristics of particular areas [39]. Geographical DFCs aim to transform entire villages, towns, cities, or regions to become more inclusive and supportive of people with dementia, potentially impacting all aspects of community life [2]. This approach emphasises the importance of adapting the physical and built environment to be more accessible and navigable for people with dementia, including clear signage, rest areas, and dementia-friendly urban design. A geographical focus also encourages involvement from various local stakeholders, such as businesses, public services, and residents, fostering a collective effort to support people with dementia. Countries like England have incorporated geographically defined DFCs into national policy [30], setting targets for their creation and establishing recognition systems, allowing for more structured implementation and evaluation. Different geographical areas may adopt diverse strategies based on their specific demographics, resources, and needs, allowing for innovation and context-specific solutions. Additionally, geographical DFCs can facilitate increased social and cultural engagement for people with dementia within their local area, helping them remain active and valued community members [34]. Defining DFCs geographically enables more straightforward evaluation of their impact on the lives of people affected by dementia within a specific area [40]. While some DFCs are also defined as communities of interest, focusing on specific groups or shared experiences rather than physical location, the geographical approach remains significant due to its comprehensive nature and ability to create tangible changes in the everyday environments where people with dementia live and interact.

This realist review will therefore offer a novel and unique contribution to the existing literature enabling a greater understanding of geographical DFCs and enable the identification of relevant interventions related to outcomes.

### Aim and objectives

The aim of this review is to create and refine a programme theory – in the form of context-mechanism-outcome (CMO) configurations – that explains how the characteristics of geographical Dementia-Friendly Communities (DFCs) interact with their social and The aim of this review is to create and refine a programme The aim of this review is to create and refine a programme The aim of this review is to create and refine a programme.

1. To identify the dominant programme theories on how geographical DFCs can be successful in improving the quality of life for people with dementia.
2. To determine the characteristics of geographical DFCs, and the social and organisational contexts that may aid or hinder their effectiveness in providing individual benefits for people with dementia.

## Methods

### Study design

A project protocol was registered with PROSPERO in March 2022 [41] with the review conducted between April 2022- February 2024. This review followed RAMESES (Realist and Meta-narrative Evidence Syntheses Evolving Standards) guidelines [42], aiming to create and refine programme theory in the form of context-mechanism-outcome (CMO) configurations.

### Step 1: scoping the literature

The first step in the review process was to define the scope of the review. This phase offered the framework and structure for examining and synthesising a variety of study findings [43]. To understand broad implementation strategies, an initial exploratory literature search was conducted. This included combining worldwide research literature to ensure a comprehensive view, grey literature such as reports and theses for practical insights, and pertinent policy papers to understand real-world applications and guidelines. Implementation strategies aim to identify and understand various methods used to implement changes effectively.

### Step 2: search methods for the review

The search strategy was developed in consultation with a subject librarian at Queen’s University Belfast. The databases searched included Cochrane Library, CINAHL, Medline, Scopus, PsychINFO and Google Scholar, as well as relevant websites such as Alzheimer’s Society to identify grey literature. The reference lists of all articles included in this review were also searched. An example of the search strategy used is shown in table 2.

**Table 2:** Example of search terms used in Medline

(((dementia OR Alzheimer*) AND (dementia friendly* OR age friendly* OR senior friendly* OR Community Network* OR social environment OR social participation OR social inclusion OR social health OR social integration) AND (sustainability OR experiences OR perceptions OR views OR feelings OR Outcomes OR Quality of life)))	

### Step 3: Selection and appraisal of articles

Covidence software [44] was utilised for the selection of articles, which automatically removed duplicate papers. All articles were reviewed by SC. PS/GM reviewed 50% of each of the articles. This ensured that two people independently and blindly reviewed each script. Any conflicts were resolved as a three-way discussion between all reviewers. The selection of articles was based on inclusion/exclusion criteria (Table 3) alongside how well they informed the programme theory. No temporal limits were applied to initial searches, however, we only searched for papers written in English language. Traditionally, realist reviews do not assess methodological quality. However, this aspect was included in this review to provide the reader with an understanding of the strength of the evidence underpinning the conclusions. The methodological quality of all included studies was assessed using JBI appraisal tools [45].

**Table 3:** Inclusion and exclusion criteria for searching

Inclusion criteria	Exclusion criteria
People living with dementia or a suspected diagnosis of dementia in a dementia friendly community	Dementia-friendly initiatives that are not clearly part of a wider dementia-friendly community.
Caregivers for those with dementia in a dementia friendly community	Dementia- friendly initiatives focusing on health and social care.
General public providing services within a dementia friendly community	Not focused on a Dementia Friendly Community

### Step 4: data extraction

A data extraction form based on the RAMESES recommendations for realist synthesis and previously used in realist reviews [46–48] was used to extract data from the included full-text papers [42] in the following areas: theoretical foundation of the intervention, participant characteristics, type of DFC intervention, how the intervention was intended to function, implementation characteristics, and contextual issues that facilitated or hindered implementation of the DFC intervention.

The review focused on theoretical foundations such as community social capital, social contagion, empowerment of PLWD, lessons from global best practices, culturally competent approaches, economic and social benefits, stakeholder involvement, and flexible adaptation of DFC models were integral. The review was also guided by strategic policies supporting DFC development and sustainability. *Context-Mechanism-Outcome (CMO*) configurations were utilised to identify *contexts* that enabled or hindered DFC initiatives, the processes or resources activated by DFCs (*mechanisms*), and the *outcomes* for people with dementia and their caregivers. Key aspects of DFCs, including physical environment adaptations, social and cultural initiatives and education and awareness programs, were systematically analysed. Implementation strategies, stakeholder engagement processes, barriers, and facilitators were also explored. The review further examined the experiences and perspectives of people living with dementia and caregivers, the impact of DFCs on caregivers, policies supporting DFCs, cultural adaptations of DFC concepts, and evaluation frameworks used to assess DFC effectiveness.

### Step 5: synthesising the evidence and drawing conclusions

#### Identification of candidate theories

A realist review focuses on the discovery, articulation, and analysis of underlying programme theories to determine if these theories are supported by the evidence [49]. Following data extraction, candidate theories were formulated, debated and reviewed with the study team. Few papers explain their programme theory; therefore, implicit theories were presumed from components of the interventions. Identifying contextual factors that aided or impeded implementation further developed each candidate theory. Candidate theories from each paper were written in the C-M-O configurations by identifying contextual factors that aid or hinder implementation.

#### Synthesis of candidate theories

The initial candidate theories were synthesised and grouped into themes relating to the context (C), mechanism (M), outcome (O), and intervention (I). All members of the research team and the study’s expert reference group discussed the relevance of the synthesised candidate theories as the programme theory was developed. The synthesised theories were combined into an overarching programme theory to indicate how geographically bounded DFC interventions may be successfully implemented in the community for people with dementia and their carers (Fig. 2).

## Results

### Study selection

**Fig. 1:**
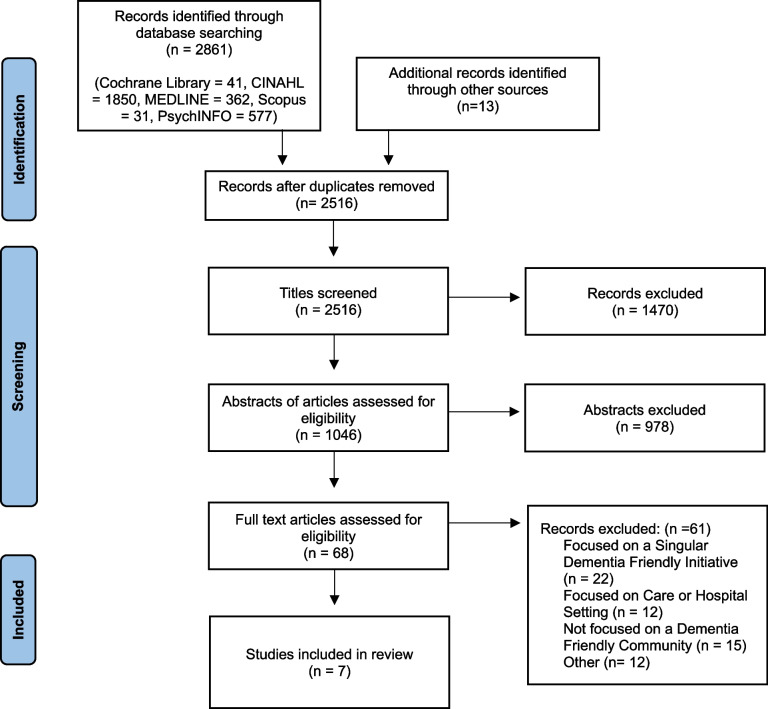
PRISMA flow diagram

The search identified 2,861 records in total (Fig. 1). After duplicates were removed a total of 2,516 papers were left. Titles and abstracts were reviewed together by S.C, P.S and G.M. Following this stage S.C. reviewed all full-text articles while P.S and G.M reviewed 50% of full-text papers. Full-text screening resulted in 68 articles for full-text review, 61 papers were excluded This was resulting in 7 papers for data extraction. Reasons for exclusion are documented in Fig. 1.

### Study characteristics (table 4)

The seven studies employed a range of methodological designs. Three studies used cross-sectional study designs [50–52]. Three articles used qualitative methodology [53–55] and one study was a mixed-methods design [56].

**Table 4:** Data extraction table for realist review

Study Information Title, author(s), year, country, and objectives	Population, setting and intervention	Design and methodological rigour (Strong/ Moderate/ Weak)	Key Results	How the intervention was thought to work	Contextual features thought to influence implementation.
Promoting cultural change towards dementia friendly communities: a multi‑level intervention in Japan Tsuda et al. [51] Tokyo: to examine from an ecological perspective the community-level effect of a multi-level DFC intervention with a particular emphasis on developing the community social capital of all the older adults living in the targeted community.	Population is currently estimated at 13,000 with 43% of the residents aged 65 and older. Older residents living in a large apartment complex in the Tokyo metropolitan area. Sample size: 2016 = 2633; 2019 = 2696 Intervention: A multi- level intervention to increase social interactions between residents with diverse backgrounds, expand the web of social networks in the community, and diffuse dementia- friendly views and attitudes.	Cross- sectional study Pre/ post- test design Rigour: Moderate	Totals of 2633 and 2696 residents completed the pre- and post-intervention surveys, respectively. The mean age of the pre-intervention respondents was 77.4 years; 45.7% lived alone and 7.7% reported living with impaired cognitive function. The proportion of men who had regular social interaction and were confident about living in their community with dementia increased significantly from 38.8 to 44.5% (*p* = 0.0080) and from 34.1 to 38.3% (*p* = 0.045), respectively. Similar significant increases were observed in the subgroup of men living with impaired cognitive function, but not in the same subgroup for women.	Providing a freely accessible communal meeting place, together with dementia awareness information and professional social support will: • Develop new social ties/ networks through social contagion • Develop inclusive societies • Provide formal support for people with dementia • Raise community awareness and knowledge • Increase PWD confidence and sense of security in the community	• People with dementia’s baseline social interaction within the community e.g. does the individual already interact and socialise within the community? • Gender of the people living with dementia in the community (women typically already have stronger social networks) • Recognisable geographical boundary of the community area. • Existing social networks within the community for PWD. • Accessible environment in a communal location
Involvement of people with dementia in raising awareness and changing attitudes in a dementia-friendly community pilot project Philipson et al. [52] Australia: to understand the extent to which education and awareness-raising activities, as well as contact with people living with dementia, would increase community understanding of dementia and reduce stigma.	Population is estimated over 21,000 people. Community members (*n* = 174) from Kiama. Kiama can be described as dispersed, consisting of a small downtown area surrounded by low-density suburbs of free-standing family homes. Kiama is an example of a ‘sea-change’ town. It is an attractive area for retirees. Intervention: A multi- component dementia- friendly intervention which was co-designed and co-facilitated to deliver dementia awareness and educational activities provided by a Dementia Advisory Group	Cross- sectional survey Rigour: Moderate	305 community members completed the pre/post-test surveys. The mean age of respondents in 2014 and 2016 was 52.34 years and 55.93 years, respectively. A large proportion of respondents knew someone with the condition in 2014 (76%) and in 2016 (78%). In 2014, 25% of respondents and in 2016, 29% of respondents reported being a current or previous carer for someone with dementia, most commonly a family member. In 2014, 30% of the samples were retired and 62% working. Whereas in 2016, almost half were retired (46%) or working (46%).	Co-designed and co-facilitated dementia awareness and educational activities; such as dementia-raising activities sharing personal experiences to 1000 community members and providing consultation services to local businesses and organisations which was provided by a Dementia Advisory Group, which also partnered with a local university to produce research which will • Support change in knowledge, attitudes, and beliefs about dementia • Increase community awareness • Reduce stigma in the community • Establish new social ties • Enhance self- efficacy	• A rural area with a recognised geographical boundary which has a smaller area/population and a strong sense of community. • Older demographic population in the community • Existing government policies within the community area and access to existing resources. • Access to existing resources e.g. advisory groups, awareness activities, diagnostic and treatment centres, community and family caregiver education and care services and political support are crucial facilitators in the successful implementation of dementia-friendly communities. • Financial support for community projects.
Asian Pacific Islander Dementia Care Network: A Model of Care for Underserved Communities Kally et al. [50] USA: to bridge the gap in services available to these three API communities in the central city area of Los Angeles.	The API population is projected to reach almost 10 million by 2020. Between 2000 and 2004, African Americans and APIs showed the greatest increase in reported deaths due to Alzheimer’s disease. In 2010, Alzheimer’s disease was the 10th leading cause of death for API men and the 6th leading cause of death for API women. Sample size: *n* = 127 caregivers Three Asian cultural groups, Chinese, Japanese and Filipino living in California. Intervention: A combined Asian Pacific Islander Dementia Care Network (APIDCN) was established to bridge the gap in services available to these three API groups. APIDCN placed stories in ethnic media, delivered general community education by care advocates and other project members, and caregiver education through written materials and through public events	Cross-sectional study Rigour: Weak	3 Key themes: • Building community capacity in each of the three targeted API communities • Building community awareness and understanding of dementia and memory loss and the importance of early diagnosis • Improved caregiver services and outcomes • Decrease in carers depression 6 months post participating in the programme.	An Asian Pacific Islander Dementia Care Network (APIDCN) placed project activities in ethnic media, delivered general community education by care advocates and other project members, and caregiver education through written materials and through public events which: • Develops community capacity • Reduces associated stigmas • Builds inclusivity within the community • Delivers direct services to caregivers	• Access to existing resources e.g. advisory groups, awareness activities, diagnostic and treatment centres, community and family caregiver education and care services and political support are crucial facilitators in the successful implementation of dementia-friendly communities. • Financial support for community projects. • Existing government policies within the community area. • Negative cultural stereotypes can also hinder implementation due to the lack of culturally appropriate services, and a lack of understanding of dementia.
Evaluation of the York Dementia Friendly Communities Programme Dean et al. [53] UK: The objective of the evaluation was to enhance learning about the process of developing and supporting DFCs, inform future work in this area and share learning with other organisations.	People working in services, businesses and projects which were used by people with dementia and their careers and supporters in the City of York. It is estimated that in 2014 there were around 2,725 people living with dementia in the city and its surrounding villages. Intervention: Dementia awareness and training were embedded into the existing life in York, through the delivery of Dementia Friends Programme funded by City of York Council and provided by local organisation Dementia Forward delivered to people living in York. This focused on intergenerational work, developed dementia friendly businesses and organisations, developed social networks through wider community support with increased training opportunities and positive media coverage.	Qualitative Rigour: Weak	• The approach in York of encouraging ‘many flowers to bloom’ has led to a range of small initiatives springing up, influenced by people with dementia and carers. • Sign-up at leadership level is evident, but the middle managers and teams which connect strategic objectives to frontline services need more support and investment • There needs to be greater focus on integration of health and social care for people with dementia.	Dementia awareness and training were embedded into the existing life in York, through the delivery of Dementia Friends Programme funded by City of York Council and provided by local organisation Dementia Forward delivered to people living in York. This focused on intergenerational work which involves children and young people engaging in work with people with dementia, developed dementia friendly businesses and organisations, developed social networks through wider community support with increased training opportunities and positive media coverage which • Grows existing networks and resources • Puts people with dementia at the heart of the project	• Population size as large groups of people can impact the intervention (Large population living in a city/ busy crowded environment) • Government support through Funding for educational activities for carers, community groups and organisations. • Not defined by a geographical boundary. • Access to existing resources e.g. advisory groups, awareness activities, diagnostic and treatment centres, community and family caregiver education and care services and political support are crucial facilitators in the successful implementation of dementia-friendly communities. • Financial support for community projects.
Dementia-friendly communities: challenges and strategies for achieving stakeholder involvement. Heward et al. [55] UK: To critically assess the challenges and strategies for achieving stakeholder involvement in DFCs.	273, 853 people from 7 localities. With 23% of the population aged 65+. The project took place in one county in the South of England, where DFCs were developed in seven localities. Two seaside towns, three market towns, one town/ borough and one borough/ seaside town were selected. This county has one of the highest proportions of the population above retirement age in the country. DF project workers in the South of England: (*n*= 74- Local Community 5- Carers 0-PWD 72- Local Organisations 559- Awareness Sessions 448- Dementia friends 19- Dementia Champions) Intervention: 7 DFCs were developed by steering group partners and 4 part-time project workers by establishing networks, involving local community, and gaining commitment from organisations in the community and environmental improvements such as providing visual information that helps PWD navigate the space	Qualitative Rigour: Weak	Challenges to achieving stakeholder involvement were identified as: • establishing networks and including people representative of the local community • involving people affected by dementia • gaining commitment from organisations. Strategies for achieving stakeholder involvement were recognised as: • a sustainable approach • spreading the word • sharing of ideas.	7 DFCs were developed across 7 localities by steering group partners and 4 part-time project workers by establishing networks, involving local community, and gaining hierarchy commitment (Commitment to the DFCs from organisations e.g. retailers and supermarkets. As businesses/ organisations had to commit to training and business action plans to be dementia- friendly. The level of involvement varied in the 7 localities due to commitment )from organisations in the community and environmental improvements such as providing visual information that helps PWD navigate the space • Which creates accessible community activities • Involves people with dementia in the community • Ensures early diagnosis • Improves the environment	• Rural geographical area where there is often a smaller population and a strong sense of community, it may be easier to engage local stakeholders. • Age of population, older aged population (retirement age) • Access to existing resources e.g. advisory groups, awareness activities, diagnostic and treatment centres, community and family caregiver education and care services and political support are crucial facilitators in the successful implementation of dementia-friendly communities. • Financial support for community projects.
National Institute for Health Research Policy Research Programme Project Dementia Friendly Communities: The DEMCOM evaluation Goodman et al. [56] UK: To identify whether dementia-friendly communities (DFCs) support people living with dementia and their carers to maintain their independence and feel valued members of their local community and, if so, which approaches have worked best and at what cost for which groups of people.	People living/ working within DFCs. 67 experts by experience (27 people living with dementia (PLWD), 31 carers, 8 former carers, 1 other) DFCs in England which are officially recognised by Alzheimer’s Society as working towards dementia-friendly status. DFCs are spread throughout the country in urban and rural areas. Intervention: Involving people with dementia as active partners in developing dementia awareness activities linked to public amenities.	Mixed Methods Rigour: Strong	The evidence from the three study phases is synthesised to develop a series of six “if then” statements to build a theory of change and logic model of how DFCs work: • **If** there is local organisations working together to promote social inclusion **then** this creates a secure basis for planning, discussion and dissemination that normalises thinking • **If** DFC collaborators understand their role, **then** this leads to activities focusing on raising awareness of the needs of people living with dementia • **If** DFC collaborators provide services for people affected by dementia **then** people living with dementia gain new networks of support and friendship • **If** people living with dementia are supported to be active partners,, **then** organisations and services learn to routinely consider the needs of people living with and affected by dementia	When PWD are involved as active partners in developing dementia awareness activities linked to public amenities it • Increases awareness and understanding • increases cultural and social engagement • increases capability of health and care services to develop services • people in organisations and services become aware of and routinely consider the needs of PWD, and stigmatising attitudes towards PWD are dispelled • PWD perceive that their needs are understood, feel included and valued within society and gain confidence to use public amenities.	• Existing government policies and support to assist the intervention. • Financial support for community projects. • Existing public awareness within the community of PWD • Recognisable geographical boundary of the community area. • Access to existing resources e.g. advisory groups, awareness activities, diagnostic and treatment centres, community and family caregiver education and care services and political support are crucial facilitators in the successful implementation of dementia-friendly communities.
Creating a Dementia Friendly Community in an African American Neighbourhood: Perspectives of People Living with Dementia, Care Partners, Stakeholders, and Community Residents Bergeron et al. [54] USA: describe perceptions of residents in a predominantly African American neighbourhood regarding building a DFC in their community which, in turn, brings us closer to understanding how to best address existing disparities.	Black/ African American community members belonging to the following groups: PLwD, family caregivers, professional/ health providers, faith- based leaders/ local business managers/ civic organisation members or neighbourhood residents. A neighbourhood of Jacksonville, Florida Intervention: A DFC toolkit based on a prior model developed by ACT. A 4-phase intervention for community engagement which includes: 1. Convening key community members to understand dementia and its implications 2. Engaging stakeholders to assess opportunities to improve dementia friendliness 3. Analysing needs that will motivate stakeholders to act 4. Prioritising an action plan	Qualitative Rigour: Weak	Three main themes emerged from the analyses, including (1) perceived needs pertaining to dementia, (2) facilitators and barriers to being dementia friendly, and (3) opportunities for the community to become more dementia friendly. Study findings highlight the unique needs of a single African American neighbourhood and the importance of culturally tailoring the dementia friendly model to diverse communities.	The DFA toolkit aims to help people with dementia remain engaged in community life by: • Educating relevant stakeholders • Improve dementia friendliness in the community • Identify specific goals, objectives and strategies to become dementia- friendly • Providing materials and resources that may be selected and adapted as appropriate, to educate recipients. • Reduce stigmatising attitudes • Create informed, safe, respectful and supportive environments.	• Disparities in Alzheimer’s disease and Alzheimer’s Disease-related diseases. • Ethnicity of community area, especially communities of colour. • Financial support for implementation. • Neighbourhood cohesion. • Ethnicity of community area. • A rural area with a recognised geographical boundary in the community. • Existing community support. • Access to existing resources.

### Methodological quality

The methodological quality of the empirical evidence in each of the seven papers included in this review was critically appraised using Joanna Briggs Institute critical appraisal tools [45]. Using the JBI tool, Goodman et al. [56] was assessed as strong, two articles were accessed as moderate [51, 52] and four were accessed as weak [50, 53–55].

### Main objectives of the studies

The included studies had three main sets of objectives: to explore the experiences of living/ working within a DFC [51, 56] and to understand how a community can become dementia-friendly [50, 52, 53, 55]. The third objective focused on the perception of residents on building a DFC in a minority area [54].

### Study populations

The studies described different types of DFCs across four continents; Asia [51] Oceania [52] North America [50, 54] and Europe [53, 55, 56]. Two studies collected data from people with dementia (*n* = 35) [54, 56]. Three studies from caregivers/ family care partners (*n* = 152) [50, 54, 55]. Four of the studies collected data from additional participants (*n* = 454). For example, community workers [52–55]. Tsuda et al. [51] categorised their participants (*n* = 2633) as older adults living in an apartment block with a mean age of 77.4, 45.7% living alone and 7.7% reported living with impaired cognitive function. Participants with a diagnosis of dementia did not disclose the clinical stage of their diagnosis.

### Characteristics of DFC interventions

All studies explored the use of dementia-friendly programmes within the community. DFC programmes involve the implementation of various person-centred approaches to the community environment to support people with dementia. The programmes identified in this realist review are not standardised interventions and do not involve a single intervention but rather a collective of different community activities interventions aided by members of the public/policymakers with ongoing input from dementia charities e.g., Alzheimer’s Society or Alzheimer’s Disease International. These programmes focus on improving the places in which people with dementia interact and live in their daily lives.

### Characteristics of DFC outcomes

DFC interventions have been shown to yield a variety of positive outcomes. These interventions have led to increased social interaction [51] among individuals living with dementia, fostering a sense of belonging and reducing social isolation [52]. Moreover, interventions promoting the involvement of people with dementia within the community have resulted in improved quality of life for people with dementia [52, 54]. DFC intervention results in improved community capacity to deliver dementia-friendly services, such as support groups and workshops, these interventions have also positively impacted caregivers by reducing depression and promoting healthy outcomes for carers [50]. Additionally, DFC interventions support people with dementia’s independence and ability to continue living in their own homes [55]. Small-scale initiatives developed by PWD and their caregivers, such as the EndAge Day and Memory Bank projects, have further enriched community engagement and encouraged participation in meaningful activities [53]. The interventions have also led to greater access to public amenities, which promotes a greater quality of life which contributes to active participation in the community and people with dementia living longer in their own homes [56].

### Candidate theories

The preliminary scoping of the literature did not identify any explicit theory underlying the implementation of DFCs for people living with dementia or their caregivers. However, common sense implicit theories were identified. It was evident that providing dementia awareness information in the community is a key component of a DFC [51–54, 56]. If dementia awareness is raised within the community, further support can be provided for people living with dementia and their caregivers which can contribute to positive changes within the environment [51, 54, 55]  and government policies [51]. This will likely encourage people with dementia and their caregivers to engage in DFCs as they will feel supported and confident in the community [51–54, 56]. In addition, this will improve the quality of life for people with dementia [56]. However, one study identified how hierarchy commitment is necessary for a business/ organisation to become dementia friendly [55]. This indicates a strong organisational commitment from the top-down of a business/ organisation. This commitment involves leaders and decision-makers at varying levels endorsing and actively participating in efforts to make the organisations more supportive of people living with dementia. This involves the business/ organisation formalising agreements to become dementia-friendly and implementing dementia-friendly action plans. This is reinforced by another study which states that communities need to prioritise an action plan when implementing a dementia friendly community [54].

### Contextual factors that help or hinder the implementation of DFC interventions

Several contextual factors were identified that help or hinder the implementation of DFC interventions for people living with dementia. The issue of having a recognisable geographical boundary for a DFC remains one of the most significant contextual factors that help the implementation of DFC interventions [51, 52, 54, 56]. However, one study states that dementia-friendly communities are not defined by a geographical boundary, they are locations where people with dementia can find their way around and feel safe in their locality/ community/ city where they can maintain their social networks, so they feel they still belong in the community [53].

Dementia-friendly communities thrive in rural areas where there is often a smaller population and a strong sense of community [52, 54] and it may be easier to engage local stakeholders [55]. Close-knit communities where people know each other well can foster greater understanding and support for people living with dementia and their caregivers, and also allow a greater opportunity for tailored and personalised interventions [54, 55].

Existing resources e.g., advisory groups, awareness activities, diagnostic and treatment centres, community and family caregiver education and care services and political support are crucial facilitators in the successful implementation of dementia-friendly communities [50, 52–56]. The presence of ample resources [50–52, 54] coupled with robust political endorsement [56], constitutes a pivotal framework for the success of such initiatives. Governmental bodies, as exemplified, play a crucial role by furnishing financial support for community projects and endorsing policies, thereby enabling a comprehensive approach to assist individuals with dementia and their caregivers (However, a range of factors that both facilitated or hindered these DFCs was also identified – for example, DFCs exhibit notable success in rural settings, as evidenced by their thriving presence in such areas [50, 52–56]. Sufficient funding is imperative for sustaining programs and services, and financial backing from governmental entities, philanthropic organisations, and local authorities becomes instrumental in meeting the expenses associated with the implementation of DFC interventions [53, 54]. Financial constraints can limit the availability of resources, services and infrastructure needed to create and sustain dementia-friendly communities [53, 54]. Political support extends beyond mere financial contributions; it catalyses the development and implementation of policies conducive to dementia-friendly practices, addressing issues like anti-discrimination measures and caregiver support. This, in turn, fosters collaboration among stakeholders [54, 55]. The establishment of policies also catalyses public awareness campaigns, aimed at mitigating associated stigmas [52, 54, 56]. By leveraging existing resources and garnering political support, communities can cultivate an environment where individuals with dementia are comprehended, esteemed, and supported. This concerted effort leads to the achievement of dementia-friendly communities, ultimately enhancing the overall quality of life for both individuals living with dementia and their caregivers.

Factors identified as hindering implementation can include the younger population’s involvement due to lack of awareness, or lack of involvement or understanding, and can indeed present some challenges in the implementation of a DFC [52, 55]. While typically younger individuals may not directly experience dementia first-hand themselves, their attitudes, understanding, and engagement in the community play a significant role in shaping the overall dementia-friendly environment. The gender of people living with dementia can also influence the implementation of dementia-friendly interventions through the concept of social contagion and the existing differences in social networks between men and women. The existing gender differences in social networks can impact the effectiveness of a DFC intervention because typically women already have stronger social networks than men [51]. Negative cultural stereotypes can also hinder implementation due to the lack of culturally appropriate services, and a lack of understanding of dementia [50]. Disparities in Alzheimer’s disease and Alzheimer’s Disease-related dementia’s create significant obstacles to the adoption of dementia-friendly communities across all communities, particularly those of colour [54].

### Synthesis of candidate theories

**Fig. 2:**
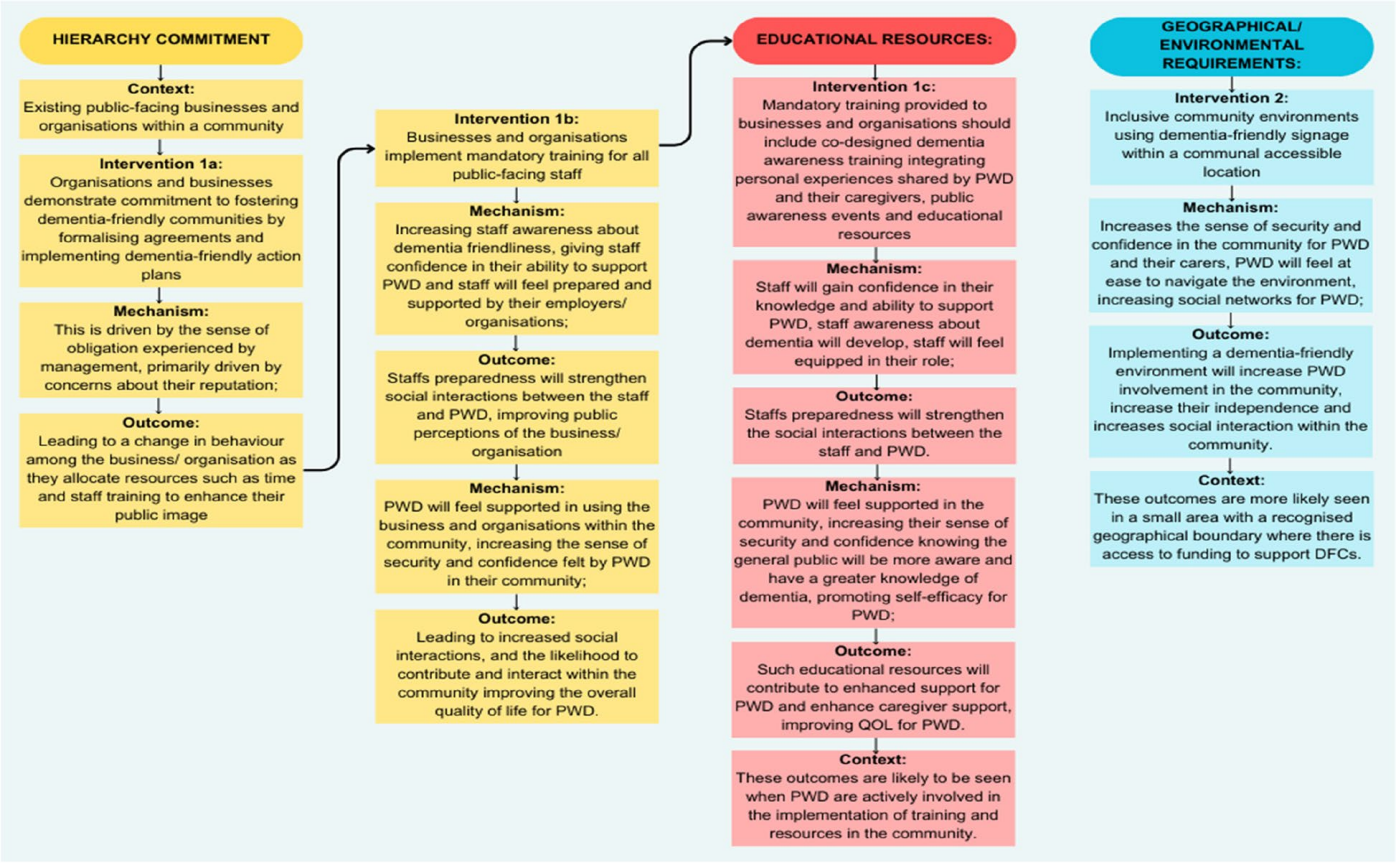
A theoretical model of how DFC interventions for people with dementia are thought to work. Legend: Theoretical model of the Context +Mechanism = Outcome (CMO) configuration. Context is shown as either helping (C+) or hindering (C-) implementation. The intervention is divided into two phases, facilitation (I1) and display (I2), activating underlying mechanisms (M) that result in improved outcomes (O)

This section explains the intervention (I), mechanism (M), and contexts (C) that are thought to produce the outcome (O) of improved quality of life (QOL) for people living with dementia, increased social interactions, support and inclusivity for people with dementia and their carers. The aim of this synthesis was to create and refine programme theory on how DFCs’ characteristics interact with their social and organisational contexts to produce desired outcomes. Figure 2 depicts the theoretical paradigm for how DFC interventions are expected to work.

The implementation of DFC interventions appeared to involve two crucial implementation phases: Hierarchy commitment (I^1a/b^)interlinked with educational resources (I^1c^) and Geographical/ environmental requirements (I^2^). Hierarchy commitment involves two sub-interventions, which are seen in existing public-facing businesses and organisations within a community (C). Organisations and businesses demonstrate a commitment to fostering dementia-friendly communities by formalising agreements and implementing dementia-friendly action plans (I^1a^). This is driven by the sense of obligation experienced by management, primarily driven by concerns about their reputation (M); leading to a change in behaviour among the business/ organisation as they allocate resources such as time and staff training to enhance their public image (O). This leads to businesses and organisations implementing mandatory training for all public-facing staff (I^1b^), which increases staff awareness about dementia friendliness (M), giving staff confidence in their ability to support PWD (M) and staff will feel prepared and supported by their employers/ organisations (M); Staffs preparedness will strengthen social interactions between the staff and PWD, improving public perceptions of the business/ organisation (O). By the same intervention, PWD will feel supported in using the business and organisations within the community (M), increasing the sense of security and confidence felt by PWD in their community (M); leading to increased social interactions, and likelihood to contribute and interact within the community improving the overall quality of life for PWD (O).

Mandatory training provided to businesses and organisations should include co-designed dementia awareness training integrating personal experiences shared by PWD and their caregivers, public awareness events and educational resources (I^1C^), staff will gain confidence in their knowledge and ability to support PWD (M), staff awareness about dementia will develop (M), staff will feel equipped in their role (M); Staffs preparedness will strengthen the social interactions between the staff and PWD (O). By the same intervention, PWD will feel supported in the community (M) increasing their sense of security and confidence knowing the general public will be more aware and have a greater knowledge of dementia (M), promoting self-efficacy for PWD (M); such educational resources will contribute to enhanced support for PWD and enhance caregiver support, improving QOL for PWD (O). These outcomes are likely to be seen when PWD are actively involved in the implementation of training and resources in the community (C).

Secondly, dementia-friendly signage creates inclusive community environments within a communal accessible location (I2) to increase the sense of security and confidence in the community for PWD and their carers (M). Further, PWD will feel at ease to navigate the environment (M), increasing social networks for PWD (M); therefore, implementing a dementia- friendly environment will increase PWD involvement in the community, increase their independence and social interaction within the community (O). These outcomes are more likely seen in a small area with a recognised geographical boundary where there is access to funding to support DFCs (C).

## Discussion

This realist review elucidates the underlying mechanisms that drive the success of DFC interventions in diverse community settings. The realist approach, rooted in understanding the interactions between contexts, mechanisms, and outcomes, allowed this review to identify the complexities of DFC interventions. The initial candidate theory emerging from the synthesis of the literature emphasised the importance of creating dementia-friendly communities to support those affected by dementia [50–56]. This theoretical model builds upon this by explicitly identifying the context and mechanisms involved in successful DFC implementation in geographical locations.

The theoretical model posits that hierarchical commitment, educational resources, and geographical/environmental requirements [50–52, 54, 56] are pivotal interventions leading to positive outcomes for individuals living with dementia. These findings extend those of the DEMCOM study’s logic model [56] by highlighting the critical role of cultural appropriateness and community structures in the success of DFCs. For instance, DFCs thrive in rural settings due to strong community ties and the utilisation of existing resources, which stimulate localised services for people with dementia [57]. However, these supports may be weakened when younger family members move away [7]. Moreover, governmental support and utilisation of existing resources significantly contribute to the facilitation of DFCs [4]. This suggests that while the DEMCOM logic model provides a robust framework, it may benefit from a more explicit integration of cultural and geographical factors. These findings challenge some conclusions of the DEMCOM study by showing that political support and financial backing, while necessary, are not sufficient on their own. The presence of culturally appropriate services and strong community engagement are equally vital. For example, the use of culturally sensitive language and involvement of community leaders were found to be critical in the API community, which was not a primary focus in the DEMCOM logic model.

The combination of context and mechanisms in this review provides an explanation as to why DFC interventions were successfully implemented. For example, recognisable geographical boundaries and rural areas [51, 52, 54, 56] facilitate the accessibility of dementia-friendly communities for people living with dementia and their carers. Government support is critical in providing resources in such areas that enable appropriate signage and environmental changes that enable engagement within this geographical boundary [50, 52, 53, 55, 56]. Effective signage tailored to individuals can create a positive environment for people living with dementia, overall improving the environment [58]. Training for the public and businesses to generate awareness with their staff supports the sustainability of dementia-friendly communities as it facilitates a widespread understanding of the disease and fosters inclusivity. Staff will also feel an increase in confidence in supporting people living with dementia in businesses within DFCs, which fosters an inclusive community that empowers people living with dementia to maintain their independence and improve their quality of life. It is acknowledged that people with dementia need to be appropriately supported and empowered to remain part of their community [59].

There are notable gaps in the evidence regarding the long-term impacts of DFCs on different demographic groups. While this study identified several immediate benefits, such as increased social engagement and reduced stigma, more longitudinal research is needed to understand the sustained impact on the quality of life and mental health outcomes for people with dementia. Additionally, there is limited evidence on the specific mechanisms through which DFCs benefit caregivers. Furthermore, factors such as the outmigration of younger individuals to larger urban areas and gender dynamics can hinder the implementation of DFCs, as evidenced by Wiersma and Denton [7] and Herron and Rosenberg [60], respectively.

This study indicates that DFCs primarily benefit people with dementia and their caregivers by enhancing social inclusion, reducing stigma, and providing culturally relevant support. In rural settings, the entire community benefits from increased awareness and support structures, contributing to a more inclusive and supportive environment for all residents. However, these findings also suggest that not all groups benefit equally. For example, in urban areas with diverse populations, the lack of culturally tailored services can limit the effectiveness of DFCs. Therefore, for DFCs to be truly effective, they must be designed with the specific needs and characteristics of the target communities in mind. According to Phillipson et al. [52], creating a model that satisfies everyone’s needs is challenging. According to Turner and Cannon [61], given their commonalities and the possibility that certain groups will have overlapping interests, it might be beneficial if projects were collaborative rather than parallel. According to research on age-friendliness in rural areas, there is variation both within and between rural communities. While younger people may leave some communities, others may see an influx of relatively wealthy retirees, which may marginalise older residents who have lived in poverty for a longer period of time [62].

The WHO [63] toolkit for dementia-friendly initiatives (DFIs) provides a valuable framework for understanding the foundational components necessary for the successful implementation of DFCs. Although our review primarily focuses on geographical DFCs, the toolkit’s recommendations can be relevant as they highlight the importance of establishing strong partnerships, engaging key stakeholders, and creating structured, well-planned initiatives that serve as the building blocks for DFCs [17, 27, 64]. DFIs and DFCs are closely related since DFIs are a part of DFCs and their results are essential to DFC support. The toolkit offers detailed guidance on how to set up DFIs, which can be seen as essential precursors to the broader goal of developing inclusive and supportive communities for individuals living with dementia.

While the research available offers significant insights into the theoretical aspects of DFC interventions, it is important to acknowledge the current lack of concrete evidence on their efficacy. Nonetheless, the realist review methodology enables us to consider the diverse perspectives of participants and stakeholders, leading to a more comprehensive understanding of the complex interplay between interventions, mechanisms, and outcomes. To ensure the sustainability of DFCs, future research should focus on the long-term impacts of existing interventions and the perspectives of decision-makers and programme creators, such as the Alzheimer’s Society. By applying the realist lens to these investigations, we can further refine our theoretical framework and identify the critical elements needed for the continued success of DFC initiatives. The realist review methodology has been instrumental in shaping a theoretical framework for the implementation of dementia-friendly communities. By acknowledging the specific contexts, identifying underlying mechanisms, and exploring outcomes, this approach moves beyond conventional systematic reviews and offers a more nuanced understanding of how DFC interventions work. While evidence on their effectiveness may still be evolving, the insights gained from this realist review contribute significantly to the growing body of knowledge, guiding the development of sustainable and effective dementia-friendly communities that truly enhance the quality of life for individuals living with dementia and their caregivers.

## Strengths and limitations

This realist review has contributed to an ever-growing evidence- base on the creation of a theoretical framework for the implementation of dementia-friendly communities, and it includes both the elements required for implementation and the underlying mechanisms that might affect outcomes. However, there was no advice on how to carry out these interventions. There is also little understanding of how the interplay between the intervention, mechanism, and setting affects people with dementia or their caregivers because DFCs were developed in various contexts and ways.

Further research looking into the sustainability of existing dementia-friendly communities is urgently needed. Future studies should also consider the lessons learned from the implementation of complex DFC interventions from people living with dementia in/and people working/volunteering within dementia-friendly communities. In acknowledging the limitations of this study, it is important to note that the existing body of literature is limited. The scarcity of relevant studies in this area may impact the generalisability of our findings and the overall programme theory. Due to the nature of the review, we could only screen English papers and therefore there may have been key literature missed. Additionally, another limitation to this study is that this review focuses solely on geographical DFCs. However, this helped to narrow the focus of this review amongst the literature.

## Conclusions

This realist review has illuminated a theoretical framework that might guide the development of geographical dementia-friendly communities for those with dementia and their caregivers. However, it has highlighted a gap in the existing literature, specifically the lack of a realist approach that explicitly theorises the specific contexts, intervention components, and resulting mechanisms. The review’s aim is to create and refine a programme theory on how to improve the experiences of living in dementia-friendly communities, which is significant for both individuals living with dementia and their caregivers. Moreover, there is a need to apply this theoretical framework to the development of geographical dementia-friendly communities, enhancing the quality of life for people living with dementia. This realist review outlines significant contextual elements, mechanisms, and outcomes in relation to geographical dementia-friendly communities which can guide future studies (Fig. 2). Future research should concentrate on building a robust body of evidence to support the sustainable implementation of dementia-friendly communities, further improving the quality of life for those diagnosed with dementia.

## Data Availability

No datasets were generated or analysed during the current study.
